# PROSPECTIVE OBSERVATIONAL STUDY OF STEP-UP DOSE TREATMENT WITH ONABOTULINUMTOXINA IN PATIENTS WITH SPASTICITY

**DOI:** 10.2340/jrm.v58.45826

**Published:** 2026-10-28

**Authors:** Masahiro ABO, Shinichiro KAWAKITA, Naohisa KIKUCHI, Nobuyuki SASAKI, Takashi SHIGEMATSU, Naho OHYANAGI, Yumi YAMAZAKI, Takao MOGI, Yoshiyuki YAMASHITA, Ai HAYASHI

**Affiliations:** 1 Department of Rehabilitation Medicine, The Jikei University Hospital, Tokyo, Japan; 2 Department of Rehabilitation Medicine, Keiju Medical Center, Nanao, Japan; 3 Department of Rehabilitation Medicine, Chiba Rehabilitation Center, Chiba, Japan; 4 Department of Rehabilitation Medicine, St. Marianna University Hospital, Kawasaki, Japan; 5 Department of Rehabilitation Medicine, Hamamatsu City Rehabilitation Hospital, Hamamatsu, Japan; 6 GlaxoSmithKline K.K., Tokyo, Japan

**Keywords:** spasticity, botulinum toxin injection, observational study

## Abstract

**Objective:**

To evaluate the safety and effectiveness of “step-up dose” onabotulinumtoxinA (401–600 units per treatment session) in patients with upper and lower limb spasticity.

**Design:**

Multicentre, prospective, observational, single-arm investigation.

**Subjects:**

A convenience sample of 109 patients with upper and lower limb spasticity scheduled to receive their first or second step-up dose onabotulinumtoxinA.

**Methods:**

The study primarily evaluated the safety of step-up dose onabotulinumtoxinA by summarizing adverse events. Effectiveness was evaluated as a secondary objective.

**Results:**

Of the safety population comprising 107 patients, 49 (45.8%) experienced at least 1 adverse event, with coronavirus disease 2019 and nasopharyngitis being the most frequent. Five patients (4.7%) reported adverse events related to onabotulinumtoxinA or injection procedure. These safety findings were generally consistent with those previously reported, while reduction of muscle tone and subsequent clinical benefits were suggested by the Modified Ashworth Scale and patient/physician-reported outcomes.

**Conclusion:**

To the best of our knowledge, this is the first study to prospectively evaluate the safety and effectiveness of onabotulinumtoxinA at doses above 400 units given simultaneously for upper and lower limb spasticity. No new safety concern was identified for this “step-up dose” treatment.

## LAY ABSTRACT

With the aim of evaluating the safety and effectiveness of onabotulinumtoxinA, a muscle relaxant, at higher doses of 401–600 units per treatment session, an observational study was conducted to follow up 109 patients with upper and lower limb spasticity (muscle stiffness) for 1 year. About half of the patients experienced at least 1 adverse event after the treatment, with coronavirus disease 2019 and common colds being the most frequent. Among these adverse events, those reported by 5 patients were judged to be side effects of onabotulinumtoxinA. These safety findings were similar to those reported in previous studies, while improvement of muscle stiffness and subsequent clinical benefits were suggested by the Modified Ashworth Scale, a method commonly used to measure stiffness, and feedback from patients and physicians. In conclusion, no new safety concern was identified for onabotulinumtoxinA at higher doses.

Spasticity is a condition caused by damage to the central nervous system, commonly resulting from chronic disorders such as stroke. It is regarded as one of the positive signs of the upper motor neuron syndrome (1), and characterized by muscle overactivity and abnormal postures in the upper and/or lower limbs. Its clinical consequences include significant interference with daily activities, unpleasant symptoms such as pain and spasms, and a substantial increase in caregiver burden as well as medical costs (2).

Treatment of spasticity comprises pharmacological therapy and surgical intervention, frequently complemented by physical therapy and therapeutic exercises. Among these approaches, the injection of type A botulinum toxin (BoNT/A) is recommended as a pharmacological treatment of choice for focal spasticity (3), and several BoNT/A products have been globally approved and utilized. OnabotulinumtoxinA (BOTOX; AbbVie Inc, North Chicago, IL, USA) is one of the most extensively studied agents, and its use for spasticity has been supported by a robust body of clinical evidence (4–6), including randomized clinical trials for upper limb spasticity (7) and lower limb spasticity (8).

Although some regional variations exist, the dose of onabotulinumtoxinA in these registration trials (400 units) has been officially approved as the upper limit for each indication, whereas the maximum dose in the simultaneous treatment of both indications has also been stipulated as 400 units. Given that spasticity often affects both the upper and lower limbs of the same patient (9–11), a large number of patients may need this combined treatment, possibly with a higher dose than that adopted for each indication individually. However, the use of onabotulinumtoxinA at doses exceeding 400 units has only been investigated in a few retrospective studies (12–14).

In Japan the maximum total body dose of onabotulinumtoxinA approved for spasticity was changed from 400 units to 600 units in November 2022. Following the approved dose increase, the present study was conducted to evaluate the safety and effectiveness of “step-up dose” onabotulinumtoxinA (401 to 600 units) in a prospective manner.

## METHODS

### Study design

This was a multicentre, prospective, observational, single-arm investigation conducted at 18 medical institutions in Japan, during the period from April 2023 to March 2025. The aim of the study was to evaluate the safety and effectiveness of step-up dose onabotulinumtoxinA, ranging from 401 to 600 units per treatment session, in patients with upper and lower limb spasticity. Additionally, describing the real-world utilization of step-up dose onabotulinumtoxinA was another objective of this study.

As this was a non-interventional study, the dosage and other parameters of onabotulinumtoxinA injection were determined at the discretion of the treating physician, in accordance with the Japanese prescribing information effective at the time this study was conducted. Hence the maximum dose was 400 units for upper limb spasticity, 300 units for lower limb spasticity, and 600 units for the combination of these 2 indications. The 48-week observation period began when the patient received their first step-up dose (baseline), and each treatment session had to be separated by an interval of 12 weeks or longer, allowing up to 5 treatment sessions during the observation period.

The study was conducted in compliance with the Declaration of Helsinki, Ethical Guidelines for Medical and Life Science Research Involving Human Subjects, and other relevant laws and regulations. The study protocol was collectively approved by the Kitamachi Clinic Ethics Review Board for all participating medical institutions. Written informed consent was obtained from all patients.

### Participants

The study enrolled patients with upper and lower limb spasticity (aged 15 years or older) scheduled to receive their first or second step-up dose of onabotulinumtoxinA. Patients expecting their second step-up dose treatment could be enrolled only when sufficient information regarding their first treatment was available, including the presence or absence of adverse events (AEs), patient- and physician-reported satisfaction with treatment, and the doses per muscle injected in their first treatment session.

Patients had to provide their informed consent before study participation. They could be withdrawn from the study at their own request, or if the physician deemed it appropriate for safety, compliance, or administrative reasons; they were also withdrawn if they received an off-label dose of onabotulinumtoxinA, or another BoNT/A formulation for spasticity.

### Outcomes

This study primarily evaluated the safety of step-up dose onabotulinumtoxinA by summarizing AEs, examining their seriousness, severity, final outcomes, and relation to onabotulinumtoxinA or injection procedure. Of all AEs observed in the study, those identified as possible distant spread of toxin (PDSOT), those related to falls and other accidents, and those related to lung function were defined as AEs of special interest, and analysed collectively as groups of similar events. As assessing the potential impact of the treatment on lung function was not feasible in an observational study, percutaneous oxygen saturation (SpO_2_) was employed as supportive bedside monitoring before and after treatment. Furthermore, results of neutralizing antibody tests were summarized if they were conducted in accordance with the Japanese prescribing information of onabotulinumtoxinA, which stipulates such tests should be performed in cases where attenuation of effect has been observed.

As the efficacy of onabotulinumtoxinA for spasticity has been evaluated in several randomized clinical trials (7, 8), this study was not designed to infer a causal effect of the treatment, and effectiveness was evaluated as a secondary objective, using measures including the Modified Ashworth Scale (MAS), Clinical Global Impression of Change (CGI-C), and patient/physician-reported satisfaction with treatment. Despite its several drawbacks including its inability to distinguish between neural and non-neural components (15, 16), the MAS is an established method to assess muscle overactivity by measuring resistance of joints to passive movement (17), and widely used in clinical practice across Japan (18). CGI-C is a 9-point Likert scale ranging from –4 (very much worse) to +4 (very much improved) (7); the physician rated the degree of change after the latest injection, considering the patient’s clinical symptoms of spasticity and their functional disability, as well as therapeutic effects and AEs of onabotulinumtoxinA. Satisfaction with treatment was assessed by the patient and the physician, each responding to a different questionnaire containing 9 and 5 questions, respectively (19). Furthermore, treatment details were recorded at each session, including muscles injected, doses per muscle, number of injection sites per muscle, and dilution of the injectate, along with concomitant medications and therapies intended for spasticity.

Data were collected using electronic case report forms. Baseline information was recorded including demographic and clinical characteristics of patients, as well as MAS scores across joints frequently treated (elbow, wrist, fingers, thumb, and ankle) and SpO_2_ levels immediately before treatment. For patients enrolled between their first and second step-up doses, baseline information and details of their first treatment were collected at the second treatment session. AEs were monitored throughout the observation period, with phone calls supplementing site visits as needed. For the purpose of assessing the impact after treatment, MAS scores, CGI-C scores, and SpO_2_ levels were considered appropriate for analysis when they were collected 4–6 weeks after a treatment session; treatment satisfaction was assessed at least 12 weeks after the injection.

### Statistical analysis

This was a descriptive study with no formal testing of hypotheses, and descriptive statistics were calculated to evaluate safety and effectiveness. Approximately 100 patients were planned to be enrolled in the safety population, which included all patients who received at least 1 treatment dose. Sample size estimation was based on an 80% probability of observing at least 1 instance of a drug-related AE with a true incidence rate of 1.8%, which was derived from a post-marketing surveillance of onabotulinumtoxinA in Japanese patients with spasticity (20). Under this assumption, 89 patients were required with accounting for an expected dropout rate of 10% during the observation period. Effectiveness outcomes were analysed for the per-protocol set, which included patients with valid effectiveness data and no major protocol deviations; patients who received off-label doses were strictly excluded to avoid any interpretation of the findings as supporting non-approved dosing. Baseline characteristics and safety outcomes were also summarized separately for patients with no prior experience (Cohort A) or a single prior experience (Cohort B) of step-up dose onabotulinumtoxinA; cohort-specific summaries were not prepared for effectiveness outcomes. No imputation was performed for missing data.

Continuous variables were summarized using the mean, standard deviation, median, minimum, and maximum values, while categorical variables were summarized as counts and percentages. For the analysis of treatment details, mode and interquartile values were also calculated to represent the commonly adopted dose distribution of onabotulinumtoxinA. AEs were coded using the Medical Dictionary for Regulatory Activities (MedDRA) Version 27.1. Statistical analyses were performed using SAS Version 9.4 (SAS Institute Inc, Cary, NC, USA).

## RESULTS

### Patient disposition and background

A convenience sample of 109 patients were screened and enrolled in the study (Fig. 1), with 84 not yet receiving their first step-up dose (Cohort A) and 25 between their first and second treatments (Cohort B). Two patients did not receive the step-up dose, which resulted in a safety population of 107 patients. Four patients were excluded from the effectiveness analysis: 3 due to the off-label administration of doses higher than those officially approved, and 1 due to the lack of effectiveness evaluation. Among the 109 enrolled patients, 92 (84.4%) completed the study; reasons for withdrawal included loss to follow-up in 8 (7.3%), investigator-assessed ineligibility in 4 (3.7%), off-label use of onabotulinumtoxinA in 3 (2.8%), and withdrawal of consent in 2 (1.8%).

**Fig. 1:**
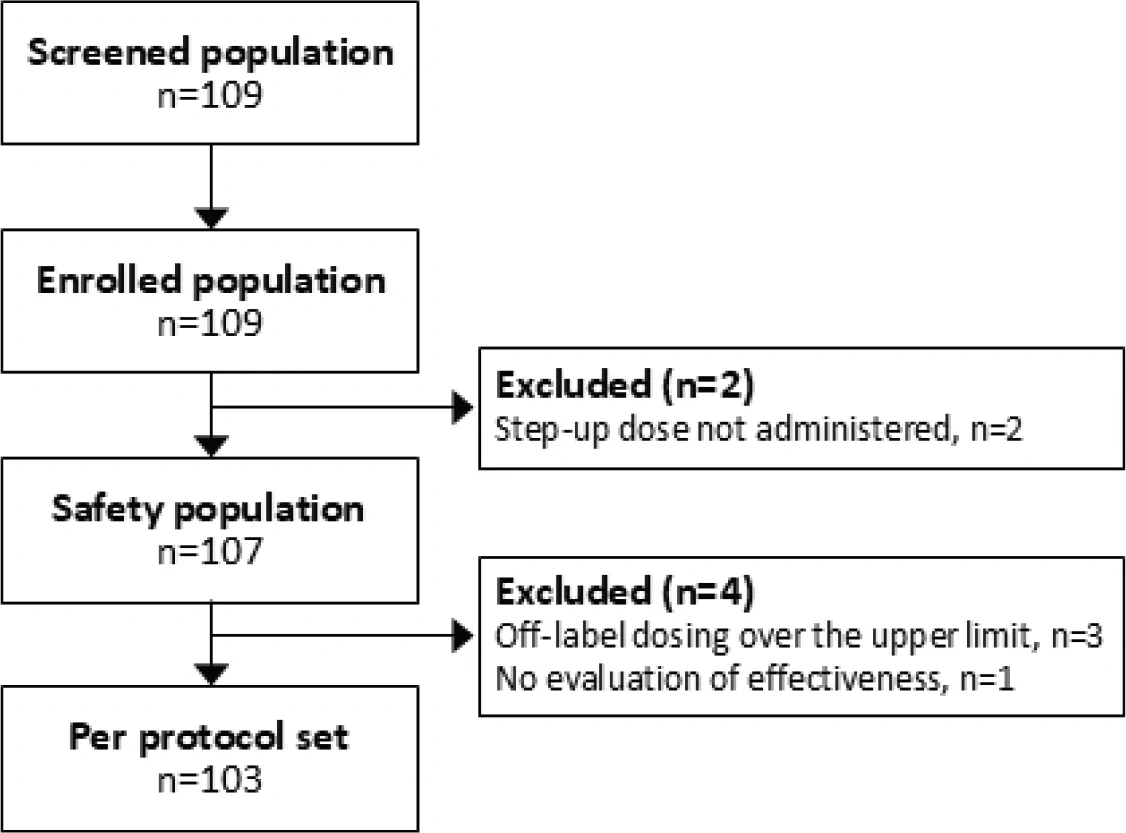
Patient disposition.

Of the 107 patients, 75 (70.1%) were male, and the mean age at baseline was 60.8 years (Table I). Stroke was the predominant aetiology of spasticity, affecting 104 patients (97.2%). Other aetiologies were identified in 4 patients, all of whom belonged to Cohort A. However, baseline characteristics were generally comparable between the 2 cohorts (Table SI). Before enrolment, 96 patients (89.7%) had the experience of receiving botulinum toxin injection for spasticity, with a mean treatment duration of 3.72 years.

**Table I:** Baseline characteristics of patients in the safety population

Characteristics	Safety population (N = 107)
Sex	
Male	75 (70.1%)
Female	32 (29.9%)
Age at baseline, years	
Mean (SD)	60.8 (10.59)
Median (Min, Max)	60.0 (40, 86)
Body mass index, kg/m^2^	
Mean (SD)	24.09 (4.001)
Median (Min, Max)	23.90 (13.9, 39.0)
Aetiology of spasticity	
Stroke	104 (97.2%)
Traumatic brain injury	2 (1.9%)
Cerebral palsy	1 (0.9%)
Spinal cord injury	1 (0.9%)
Others	0 (0.0%)
Duration of aetiology, years	
Mean (SD)	8.17 (8.315)
Median (Min, Max)	5.75 (0.9, 68.8)
History of botulinum toxin injection	
Yes	96 (89.7%)
No	11 (10.3%)
Duration of botulinum toxin injection, years	
Mean (SD)	3.72 (3.216)
Median (Min, Max)	2.90 (0.2, 12.4)

One post-stroke patient also had a history of spinal cord injury, resulting in an apparent total of 108 patients.

Max: maximum; Min: minimum; SD: standard deviation.

### Treatment details

In total, 107 patients received onabotulinumtoxinA across 412 treatment sessions. The number of patients during Treatment Cycles 1–5 was 107, 106, 95, 80, and 24, respectively. The mean interval between treatment sessions was 15.22 weeks (range, 12–44 weeks). Regarding dilution of toxin, the median volume of injectate containing 100 units of onabotulinumtoxinA was 4.0 mL, with an interquartile range of 2.0–4.0 mL for the upper limb and 2.5–4.0 mL for the lower limb.

Across the 5 treatment cycles, the median and mode of the total body dose were consistently 600 units, with an interquartile range of 500–600 units (Table II). Doses per limb also remained stable, with median doses of 300 units for the upper limb and 250–275 units for the lower limb; the mode was invariably 300 units for each of them. More treatment details, such as muscles injected and doses per muscle, can be found in Tables SII–SXIV.

**Table II:** Overall dosing of onabotulinumtoxinA across treatment cycles

Item	Treatment Cycle 1 (N = 107)	Treatment Cycle 2 (N = 106)	Treatment Cycle 3 (N = 95)	Treatment Cycle 4 (N = 80)	Treatment Cycle 5 (N = 24)
Total (upper & lower limbs), units					
Mean (SD)	553.88 (58.420)	548.35 (73.620)	538.68 (85.269)	542.31 (76.099)	555.63 (76.745)
Median (Q1, Q3)	600 (500, 600)	600 (500, 600)	600 (500, 600)	600 (500, 600)	600 (500, 600)
Mode	600	600	600	600	600
Upper limb(s), units					
Mean (SD)	311.54 (65.034)	305.24 (62.594)	295.48 (66.491)	293.00 (73.474)	311.46 (69.462)
Median (Q1, Q3)	300 (300, 370)	300 (300, 350)	300 (280, 325)	300 (275, 325)	300 (287.5, 350)
Mode	300	300	300	300	300
Lower limb(s), units					
Mean (SD)	242.34 (67.009)	248.33 (63.373)	251.61 (59.728)	249.31 (62.139)	254.78 (43.887)
Median (Q1, Q3)	250 (200, 300)	275 (200, 300)	275 (200, 300)	275 (200, 300)	250 (200, 300)
Mode	300	300	300	300	300

Most patients had their upper and lower limbs treated at the same treatment session, with a few exceptions: no injection was given for the upper limb in a total of 2 patients (1 in Treatment Cycles 2 and 3, respectively), and for the lower limb in a total of 4 patients (1 in Treatment Cycles 2 and 5, respectively, and 2 in Treatment Cycle 3).

Q1: first quartile; Q3: third quartile; SD: standard deviation.

During the post-injection period, 93 patients (86.9% of the safety population) underwent 1 or more modalities of adjunctive therapy in various situations categorized as outpatient (37.4%), inpatient (15.9%), day care (40.2%), home visit (29.9%), self-training (7.5%), and others (1.9%), with some patients falling into more than 1 category. Most frequently utilized modalities included stretching/range of motion exercises (68.2%), functional training (47.7%), splints/orthotics (45.8%), and muscle strengthening (31.8%). Concomitant medications for spasticity were used by 27 patients (25.2%), with tizanidine hydrochloride (8.4%) and baclofen (6.5%) being the most common.

### Safety

A total of 49 patients (45.8%) experienced at least 1 AE, with coronavirus disease 2019 (COVID-19; 9.3%) and nasopharyngitis (6.5%) being the most frequent (Table III). The incidence per cycle was highest in Treatment Cycle 1 (20.6%) and lowest in Treatment Cycle 4 (11.3%), while no clear pattern was observed between the total body dose and the incidence (Table SXV). The incidence of AEs was numerically higher in males (49.3%) compared with females (37.5%), and in patients younger than 65 years (47.8%) compared with those aged 65 years or older (42.1%). Cohort A also exhibited a numerically higher AE incidence (48.8%) than did Cohort B (36.0%); the incidence across Treatment Cycles 1–4 was 25.6%, 14.8%, 13.7%, and 12.7% for Cohort A, and 4.0%, 28.0%, 22.7%, and 5.9% for Cohort B.

**Table III:** Adverse events across treatment cycles

Type	Total (N = 107)	Treatment Cycle 1 (N = 107)	Treatment Cycle 2 (N = 106)	Treatment Cycle 3 (N = 95)	Treatment Cycle 4 (N = 80)
Any event	49 (45.8%)	22 (20.6%)	19 (17.9%)	15 (15.8%)	9 (11.3%)
COVID-19	10 (9.3%)	5 (4.7%)	1 (0.9%)	2 (2.1%)	2 (2.5%)
Nasopharyngitis	7 (6.5%)	2 (1.9%)	1 (0.9%)	2 (2.1%)	2 (2.5%)
Cellulitis	4 (3.7%)		2 (1.9%)	2 (2.1%)	
Periarthritis	3 (2.8%)	1 (0.9%)	1 (0.9%)	1 (1.1%)	
Cerebral haemorrhage	2 (1.9%)		2 (1.9%)		
Cerebral infarction	2 (1.9%)	1 (0.9%)		1 (1.1%)	
Vertigo positional	2 (1.9%)		2 (1.9%)		
Eczema	2 (1.9%)	1 (0.9%)	1 (0.9%)		
Back pain	2 (1.9%)	1 (0.9%)	1 (0.9%)		
Asthenia	2 (1.9%)	2 (1.9%)			
Pyrexia	2 (1.9%)		1 (0.9%)	1 (1.1%)	
Femoral neck fracture	2 (1.9%)		1 (0.9%)		1 (1.3%)
Contusion	2 (1.9%)	2 (1.9%)			

Those observed in more than 1 case are shown here.

COVID-19: coronavirus disease 2019.

Five patients (4.7%) reported AEs with a causal relationship to onabotulinumtoxinA or injection procedure as determined by the investigators: asthenia in 2 patients, muscular weakness, cellulitis, and haemorrhage subcutaneous in 1 patient each. The incidence was 4.9% (4/82) and 4.0% (1/25) in Cohorts A and B, respectively, with only cellulitis observed in the latter. Two of these AEs were serious (asthenia and cellulitis in 1 patient each); none led to death or treatment discontinuation. As for the final outcomes confirmed during the observation period, cellulitis was “resolving” 33 days after the injection, muscular weakness was “not resolved” 168 days after the injection, and the other 3 were “resolved”.

Serious AEs were observed in 18 patients (16.8%), with incidences of 18.3% (15/82) and 12.0% (3/25) in Cohorts A and B, respectively. Two patients died, 1 due to lung neoplasm malignant and thyroid cancer, and the other due to cerebral haemorrhage. In addition to these fatal cases, 2 patients discontinued the treatment due to serious AEs, 1 with cerebral haemorrhage, and the other with pseudomembranous colitis, pneumonia aspiration, and septic shock. No patient discontinued the treatment due to non-serious AEs. Furthermore, 6 patients (5.6%) underwent a dose reduction due to AEs including asthenia in 2 patients, muscular weakness, torticollis, dermatitis, and oedema peripheral in 1 patient each; asthenia (in both patients) and muscular weakness were found to be related to onabotulinumtoxinA.

Of the 40 events pre-identified for the PDSOT analysis, 4 were observed in 1 patient each: pneumonia aspiration, constipation, muscular weakness, and urinary retention (Table SXVI). A thorough medical review revealed that muscular weakness was localized to the injected limb, and the other 3 were confounded by ongoing medical history or concomitant medication, demonstrating no medically confirmed case of PDSOT in the study. In addition, based on the pre-specified list of AEs, our analysis identified 7 AEs (in 8 patients) related to falls and other accidents, as well as 2 AEs related to lung function (Table SXVI). Of these AEs of special interest, none were related to onabotulinumtoxinA or injection procedure except the localized muscular weakness.

As for SpO_2_ across treatment cycles (Table SXVII), median values were 97–98% both before and after treatment; the minimum values were 94–95% before treatment and 93–94% after treatment, indicating that no patient recorded values below 90%. The median change was 0% across treatment cycles, and the mean change ranged from –0.2% to 0.1%.

No patient underwent any neutralizing antibody test during the observation period.

### Effectiveness

MAS scores were coded from 0, 1, 1+, 2, 3, or 4 to 0, 1, 2, 3, 4, or 5, respectively, for the purpose of analysis. Median values before treatment were 3 (after coding) except for the thumb in Treatment Cycles 2–4, and generally decreased to 2 or lower after treatment, except for the ankle in Treatment Cycle 3 (Table IV). Overall, median changes indicated a decrease of 1 point at these joints in each of the 4 treatment cycles.

**Table IV:** Changes in Modified Ashworth Scale (MAS) scores across treatment cycles

Change	Treatment Cycle 1 Median (Min, Max)	Treatment Cycle 2 Median (Min, Max)	Treatment Cycle 3 Median (Min, Max)	Treatment Cycle 4 Median (Min, Max)
Elbow, *n*	57	58	47	40
Before treatment	3 (1, 4)	3 (1, 4)	3 (1, 4)	3 (1, 4)
After treatment	2 (0, 4)	2 (0, 4)	2 (1, 3)	2 (1, 4)
Change	–1 (–3, 0)	–1 (–3, 1)	–1 (–3, 1)	–1 (–2, 1)
Wrist, *n*	57	53	43	34
Before treatment	3 (1, 4)	3 (1, 4)	3 (1, 4)	3 (1, 4)
After treatment	2 (0, 4)	2 (0, 3)	2 (1, 4)	2 (1, 4)
Change	–1 (–4, 1)	–1 (–3, 1)	–1 (–3, 1)	–1 (–2, 1)
Fingers, *n*	58	57	50	41
Before treatment	3 (1, 4)	3 (0, 4)	3 (0, 4)	3 (1, 4)
After treatment	2 (0, 4)	2 (0, 4)	1 (0, 4)	2 (1, 4)
Change	–1 (–3, 1)	–1 (–3, 2)	–1 (–3, 1)	–1 (–2, 1)
Thumb, *n*	24	40	36	28
Before treatment	3 (0, 5)	2 (1, 4)	2 (0, 4)	2 (1, 4)
After treatment	1.5 (0, 4)	1.5 (0, 4)	1 (0, 3)	2 (0, 3)
Change	–2 (–3, 2)	–1 (–3, 1)	–1 (–3, 0)	–1 (–2, 2)
Ankle, *n*	62	67	55	45
Before treatment	3 (0, 5)	3 (0, 4)	3 (1, 4)	3 (1, 4)
After treatment	2 (0, 4)	2 (0, 4)	3 (1, 4)	2 (0, 4)
Change	–1 (–2, 2)	–1 (–2, 1)	0 (–2, 2)	–1 (–2, 3)

Patients with both pre- and post-treatment values were included in the analysis for each treatment cycle.

MAS scores of 0, 1, 1+, 2, 3, and 4 were coded as 0, 1, 2, 3, 4, and 5, respectively, for tabulation.

CGI-C scores also demonstrated generally positive outcomes. Although no patient was rated as +4 (very much improved), the proportion of patients showing scores of improvement ranging from +3 (much improved) to +1 (minimally improved) after Treatment Cycles 1–4 was 88.7% (55/62), 81.4% (57/70), 78.9% (45/57), and 83.0% (39/47), respectively.

Across the 4 treatment cycles, 56.3% (162/288) of patients responded that they were either satisfied or extremely satisfied with how their most recent onabotulinumtoxinA treatment helped their spasticity (Fig. 2A). When asked if they would continue onabotulinumtoxinA to treat their spasticity, 87.5–94.7% of patients answered, “Probably Yes” or “Yes, Definitely” across treatment cycles (Fig. 2I).

**Fig. 2:**
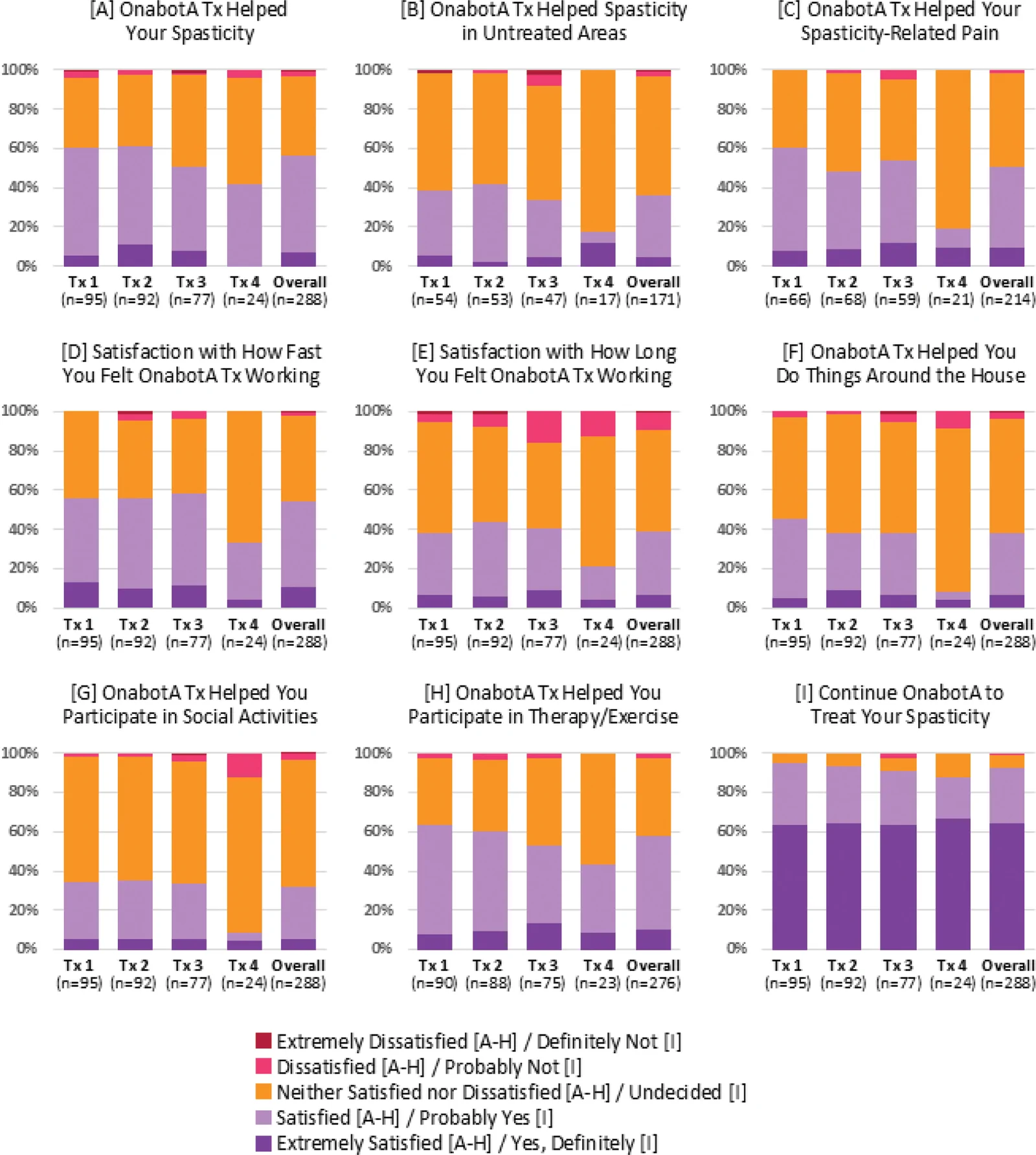
Patient-reported satisfaction with onabotulinumtoxinA (OnabotA) treatment (Tx).

As compared with ratings by patients, physician-reported satisfaction with onabotulinumtoxinA treatment was generally higher (Fig. S1). All physicians answered “Probably Yes” or “Yes, Definitely” when asked if they would continue onabotulinumtoxinA to manage their patient’s spasticity (Fig. S1E).

## DISCUSSION

In this observational study, the safety and effectiveness of onabotulinumtoxinA at doses up to 600 units given simultaneously in upper and lower limbs were prospectively evaluated for the first time, as far as we know, in more than 100 patients with spasticity.

The incidence of AEs was 45.8%, which was apparently elevated by the presence of COVID-19, a factor not reported in previous studies. The AE incidence did not show any sign of increase as the treatment was repeated up to 4 times, and demonstrated no clear pattern with the total body dose, suggesting no specific risk associated with repeated injections of step-up dose onabotulinumtoxinA. Four AEs in 5 patients were considered related to onabotulinumtoxinA or injection procedure, most of which have been commonly observed in the medical literature. Eighteen patients (16.8%) experienced serious AEs, leading to death in 2 patients and the discontinuation of treatment in another 2; none of these events were related to onabotulinumtoxinA or injection procedure. Overall, these safety profiles are generally consistent with those reported in previous studies (7, 19, 21), and no new safety concern was suggested in the present study.

Three groups of AEs were examined as AEs of special interest in this study: those identified as PDSOT, those related to falls and other accidents, and those related to lung function. PDSOT is defined as a possible pharmacologic effect of botulinum toxin at sites non-contiguous and distant from the site of injection, and injection of higher doses may be regarded as one of its risk factors (22, 23). However, no AE was medically confirmed as a result of toxin spread in this study. As for the other 2 groups of AEs, falls can be a detrimental consequence of spasticity (24), as well as a safety concern in the botulinum toxin treatment of spasticity (7), while possible effects on lung function may warrant caution in the use of botulinum toxin for upper limb spasticity (25). In this study, however, none of these AEs were considered related to onabotulinumtoxinA or injection procedure. Furthermore, although standard lung function tests were not feasible in this observational study, SpO_2_ values remained stable throughout the observation period. Again, the analysis of these AE categories, along with the supportive bedside monitoring of a respiratory indicator, revealed no new safety concern associated with the step-up dose treatment.

Use of higher doses may also be associated with an increased risk of developing neutralizing antibodies (26). The Japanese prescribing information of onabotulinumtoxinA stipulates that an antibody assay should be performed if attenuation of effect has been observed; however, no patient enrolled in this study underwent such testing during the observation period. This result was consistent with the improvement of muscle overactivity as measured by the MAS. In each of the 4 treatment cycles, evaluation of MAS scores at 5 joints generally revealed a median decrease of 1 point, which is often regarded as a clinically meaningful improvement of spasticity (27–29). The amelioration of spasticity was also reflected in CGI-C scores, with the proportion of patients showing improvement ranging from 78.9% to 88.7% across the 4 treatment cycles.

The effectiveness of onabotulinumtoxinA was also suggested by patient-reported treatment satisfaction, with “Satisfied” and “Extremely Satisfied” accounting for more than half of the responses regarding its effect on spasticity, spasticity-related pain, and the facilitation of therapy and exercises. Satisfaction with the duration of effect was somewhat lower, possibly indicating unmet needs due to the nature of this treatment, which requires repeated injections every 3 to 4 months (30). Furthermore, a trend of decreased satisfaction was noted after the fourth treatment session during the observation period, and the interpretation of this finding warrants caution. Because all patients in this study were to be observed for the same length of time (48 weeks), those who underwent the fourth treatment during the observation period naturally received repeated injections at shorter intervals than those who had fewer treatment sessions; this may suggest a possibility that they were less responsive to treatment, more susceptible to symptom recurrence, or experienced a wearing-off effect at the end of the treatment cycle. As treatment satisfaction was assessed at least 12 weeks after the injection, the outcome may have been worse in patients with shorter duration of effect. In other words, the decline in satisfaction may have been caused by patient factors, rather than by the repetition of treatment.

Patient-reported satisfaction was generally lower than physician-reported satisfaction, a finding consistent with results from previous studies (19, 21). This disparity may be partly explained by patients’ unrealistic expectations of a new treatment (31), as they were newly introduced to step-up dose onabotulinumtoxinA. Additionally, insufficient knowledge and/or information regarding the treatment may be another reason (31). Patients may not clearly understand which of their clinical issues can be addressed by this particular treatment, or they may not be able to recognize post-treatment changes if they do not know what to expect. Therefore, close communication between the patient and the physician, along with proper goal and expectation setting, may facilitate their understanding of treatment outcomes and thus contribute to enhanced treatment satisfaction.

### Limitations

The present study has several limitations. Due to its observational nature, details of medical management were determined at the discretion of the treating physician, which may have limited the availability of effectiveness evaluations at optimal time points, or confounded treatment outcomes across participating institutions. No statistical tests were performed to evaluate treatment outcomes, as the study was not designed for hypothesis testing, and effectiveness was predominantly assessed using subjective measures, with no explicit evaluation of patients’ functional status. Improvement in these outcomes should be interpreted as a consequence of total management including adjunctive therapy and/or medications; it should be noted that 93 patients (86.9% of the safety population) underwent 1 or more modalities of adjunctive therapy, and concomitant medications for spasticity were used by 27 patients (25.2%). Furthermore, satisfaction with treatment was assessed using questionnaires adapted from an international observational study (19, 21), but neither the English nor the Japanese version has undergone formal validation.

Patient characteristics may also limit the generalizability of study results. Despite our intention to enrol spasticity patients of various aetiologies, more than 90% had post-stroke spasticity, possibly reflecting a lack of centres specializing in conditions such as cerebral palsy or spinal cord injury. Additionally, this was a convenience sample of patients with spasticity, most of whom had a history of botulinum toxin injections, and this background may have “enriched” the population by excluding non-responders and patients experiencing AEs; it should be noted that patients who discontinue the treatment because of AEs or lack of effectiveness are inherently underrepresented. Of note, treatment outcomes may vary depending on factors such as aetiology or severity of spasticity, and for treatment-naive patients, initiating the treatment with step-up doses generally cannot be recommended.

One more limitation is that to secure an adequate number of patients, the study protocol allowed for the inclusion of those scheduled to receive their second step-up dose onabotulinumtoxinA (Cohort B), and only 1 AE (COVID-19) was recorded in this group after their first step-up dose treatment. Given that baseline characteristics were generally comparable between the cohorts, as was the incidence of AEs related to onabotulinumtoxinA or injection procedure, it seems unlikely that our analysis failed to detect negative outcomes caused by onabotulinumtoxinA in Cohort B. However, some AEs may have gone unnoticed after the first treatment because of their apparent lack of causative relation to the treatment.

Despite these limitations, the study has yielded valuable findings to inform the clinical practice of spasticity treatment. In this study, the safety and effectiveness of onabotulinumtoxinA injection was systematically evaluated at a median dose of 600 units, with an interquartile range of 500–600 units, in a population predominantly comprising post-stroke patients in the chronic phase, which is obviously the largest group of spasticity patients indicated for botulinum toxin injection. Our analysis also demonstrated how these step-up doses were utilized in clinical practice, including muscles selected for injection, doses chosen for each muscle, and the combination of muscles treated at each joint (see Tables SII–SXIV). This information is expected to help physicians optimize the treatment to better suit each of these highly heterogeneous patients.

### Conclusion

No new safety concern was identified for onabotulinumtoxinA at doses above 400 units given simultaneously for upper and lower limb spasticity. To the best of our knowledge, this is the first study to prospectively evaluate this “step-up dose” treatment, and the treatment details reported here will likely assist physicians in determining clinical strategies for managing spasticity.

## Supplementary Material




