# Supplementary material for: PROSPECTIVE OBSERVATIONAL STUDY OF STEP-UP DOSE TREATMENT WITH ONABOTULINUMTOXINA IN PATIENTS WITH SPASTICITY
Source: J Rehabil Med. 2026 Oct 28;58:45826. doi: 10.2340/jrm.v58.45826 (PMC13722992; doi:10.2340/jrm.v58.45826)

Fig. S1. Physician-reported satisfaction with onabotulinumtoxinA treatment.
OnabotA: onabotulinumtoxinA; Tx: treatment.

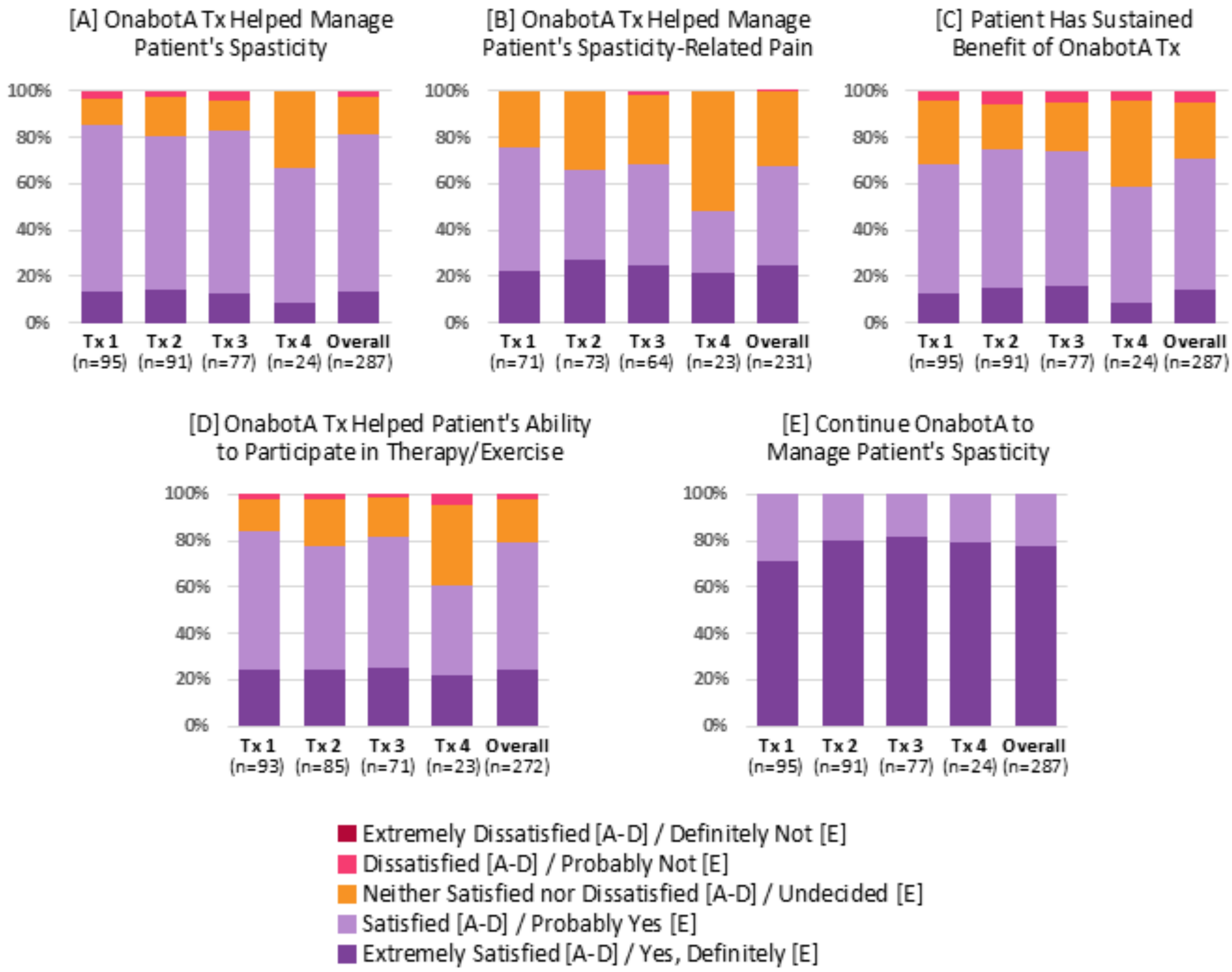

Supplement: Supplementary file 1 [file JRM-58-45826-s1.pdf]
