# Supplementary material for: PROSPECTIVE OBSERVATIONAL STUDY OF STEP-UP DOSE TREATMENT WITH ONABOTULINUMTOXINA IN PATIENTS WITH SPASTICITY
Source: J Rehabil Med. 2026 Oct 28;58:45826. doi: 10.2340/jrm.v58.45826 (PMC13722992; doi:10.2340/jrm.v58.45826)
Supplement: Supplementary file 2 [file JRM-58-45826-s2.pdf]

Table SI. *Baseline characteristics of patients in the safety population*

Characteristics	Total (N=107)	Cohort A (N=82)	Cohort B (N=25)
Sex			
Male	75 (70.1%)	58 (70.7%)	17 (68.0%)
Female	32 (29.9%)	24 (29.3%)	8 (32.0%)
Age at baseline, years			
Mean (SD)	60.8 (10.59)	61.7 (10.55)	57.7 (10.34)
Median (Min, Max)	60.0 (40, 86)	61.5 (40, 86)	59.0 (41, 78)
Body mass index, kg/m^2			
Mean (SD)	24.09 (4.001)	24.20 (3.946)	23.70 (4.235)
Median (Min, Max)	23.90 (13.9, 39.0)	23.75 (13.9, 39.0)	24.90 (14.2, 28.7)
Etiology of spasticity			
Stroke	104 (97.2%)	79 (96.3%)	25 (100.0%)
Traumatic brain injury	2 (1.9%)	2 (2.4%)	0 (0.0%)
Cerebral palsy	1 (0.9%)	1 (1.2%)	0 (0.0%)
Spinal cord injury	1 (0.9%)	1 (1.2%)	0 (0.0%)
Others	0 (0.0%)	0 (0.0%)	0 (0.0%)
Duration of etiology, years			
Mean (SD)	8.17 (8.315)	8.13 (8.965)	8.32 (5.839)
Median (Min, Max)	5.75 (0.9, 68.8)	5.42 (0.9, 68.8)	7.25 (1.2, 25.1)
History of botulinum toxin injection			
Yes	96 (89.7%)	73 (89.0%)	23 (92.0%)
No	11 (10.3%)	9 (11.0%)	2 (8.0%)
Duration of botulinum toxin injection, years			
Mean (SD)	3.72 (3.216)	3.55 (3.416)	4.24 (2.468)
Median (Min, Max)	2.90 (0.2, 12.4)	2.60 (0.2, 12.4)	4.42 (0.6, 10.2)

Max: maximum; Min: minimum; SD: standard deviation.

One post-stroke patient also had a history of spinal cord injury, resulting in an apparent total of 108 patients.

Table SII. *Doses per joint*

	No. patients treated	No. treatment sessions	Mean dose (SD), units	Median dose (Q1, Q3), units	Mode, units
Upper limb					
Shoulder	75	252	85.40 (60.817)	72.5 (50, 100)	50
Elbow	100	366	98.27 (44.459)	100 (60, 120)	100
Forearm (pronation)	54	159	43.87 (23.160)	40 (25, 50)	50
Wrist	93	317	72.37 (34.385)	60 (50, 100)	50
Fingers	101	358	80.34 (46.666)	67.5 (50, 100)	50
Thumb	73	223	35.49 (16.233)	30 (25, 50)	50
Lower limb					
Hip	16	47	56.28 (31.389)	50 (30, 80)	30
Knee	35	124	83.02 (35.930)	77.5 (50, 100)	100
Ankle	105	399	188.82 (69.691)	200 (140, 250)	200
Toes	71	223	57.98 (32.514)	50 (40, 75)	50

Q1: first quartile; Q3: third quartile; SD: standard deviation.

Table SIII. *Doses per muscle in the upper limb*

Joints	Muscles	No. patients treated	No. treatment sessions	Mean dose (SD), units	Median dose (Q1, Q3), units	Mode, units
Shoulder	Pectoralis major	69	232	54.89 (21.943)	50 (42.5, 60)	50
	Latissimus dorsi	19	66	53.94 (14.156)	50 (50, 50)	50
	Teres major	14	44	42.16 (21.577)	40 (25, 50)	50
	Subscapularis	9	24	27.08 (5.299)	25 (25, 30)	25
	Deltoid	1	2	60.00 (28.284)	60 (40, 80)	-
	Supraspinatus	1	1	40	40 (40, 40)	40
	Others	12	25	102.40 (123.044)	50 (40, 50)	50
Elbow	Biceps brachii	94	324	61.00 (23.649)	50 (50, 75)	50
	Brachialis	64	197	45.70 (17.954)	50 (30, 50)	50
	Brachioradialis	37	87	36.49 (16.014)	30 (25, 50)	25
	Triceps brachii	27	74	54.35 (23.784)	50 (50, 50)	50
Forearm (pronation)	Pronator teres	54	159	42.14 (19.328)	40 (25, 50)	50
	Pronator quadratus	3	8	34.38 (12.939)	25 (25, 50)	25
Wrist	Flexor carpi radialis	88	297	49.11 (14.766)	50 (50, 50)	50
	Flexor carpi ulnaris	59	171	42.46 (16.027)	50 (25, 50)	50
	Palmaris longus	10	23	47.61 (7.365)	50 (40, 50)	50
Fingers	Flexor digitorum superficialis	98	338	54.67 (24.752)	50 (45, 50)	50
	Flexor digitorum profundus	53	169	50.62 (24.971)	50 (40, 50)	50
	Lumbricals	15	44	33.86 (17.416)	25 (20, 50)	50
	Dorsal interossei	5	12	17.50 (3.371)	15 (15, 20)	15
	Extensor digitorum	1	1	25	25 (25, 25)	25
Thumb	Flexor pollicis longus	63	176	31.18 (13.588)	25 (20, 50)	25
	Flexor pollicis brevis	5	5	33.00 (17.176)	30 (25, 50)	50
	Adductor pollicis	27	75	27.03 (15.573)	20 (15, 50)	50
	Opponens pollicis	6	12	19.58 (8.107)	20 (15, 27.5)	15

Q1: first quartile; Q3: third quartile; SD: standard deviation.

Table SIV. *Doses per muscle in the lower limb*

Joints	Muscles	No. patients treated	No. treatment sessions	Mean dose (SD), units	Median dose (Q1, Q3), units	Mode, units
Hip	Psoas major	2	2	40.00 (14.142)	40 (30, 50)	-
	Iliacus	1	1	100	100 (100, 100)	100
	Quadratus lumborum	1	4	80.00 (0.000)	80 (80, 80)	80
	Tensor fasciae latae	2	4	40.00 (23.094)	40 (20, 60)	20
	Sartorius	2	2	22.50 (3.536)	22.5 (20, 25)	-
	Adductor longus	7	21	45.71 (28.648)	30 (25, 50)	30
	Adductor magnus	7	13	57.69 (31.399)	50 (30, 100)	50
	Pectineus	2	2	60.00 (14.142)	60 (50, 70)	-
	Gracilis	1	4	27.50 (5.000)	30 (25, 30)	30
Knee	Rectus femoris	6	10	32.00 (16.533)	25 (25, 50)	25
	Vastus lateralis	3	4	40.00 (20.000)	50 (30, 50)	50
	Vastus intermedius	1	1	150	150 (150, 150)	150
	Biceps femoris	25	72	52.76 (22.268)	50 (40, 50)	50
	Semitendinosus	23	66	48.41 (16.505)	50 (40, 50)	50
	Semimembranosus	23	64	41.73 (13.204)	50 (30, 50)	50
Ankle	Gastrocnemius	103	354	97.57 (38.354)	100 (75, 100)	100
	Soleus	91	317	72.73 (26.853)	75 (50, 100)	50
	Tibialis posterior	80	261	62.91 (27.910)	50 (50, 75)	50
	Tibialis anterior	13	34	38.97 (18.248)	30 (25, 50)	25
Toes	Flexor digitorum longus	64	189	44.58 (18.803)	50 (30, 50)	50
	Flexor digitorum brevis	8	21	38.57 (10.856)	40 (30, 50)	50
	Flexor hallucis longus	40	91	34.12 (16.066)	25 (20, 50)	50
	Flexor hallucis brevis	3	6	31.67 (10.801)	25 (25, 40)	25
	Extensor digitorum brevis	1	1	10	10 (10, 10)	10
	Extensor hallucis longus	8	10	39.00 (17.764)	40 (25, 50)	50

Q1: first quartile; Q3: third quartile; SD: standard deviation.

Table SV. *Frequently treated combinations of shoulder muscles (75 patients, 252 treatment sessions)*

	Total dose	PM dose	LD dose	TM dose	SS dose
PM alone (48 patients, 140 treatment sessions)					
Median (Q1, Q3), units	50 (40, 55)	50 (40, 55)	-	-	-
Mode, units	50	50	-	-	-
PM+LD (11 patients, 42 treatment sessions)					
Median (Q1, Q3), units	100 (100, 150)	50 (50, 100)	50 (50, 50)	-	-
Mode, units	100	50	50	-	-
PM+LD+TM (7 patients, 16 treatment sessions)					
Median (Q1, Q3), units	200 (165, 200)	55 (50, 100)	50 (50, 60)	50 (50, 50)	-
Mode, units	200	50	50	50	-
PM+TM+SS (5 patients, 13 treatment sessions)					
Median (Q1, Q3), units	90 (80, 100)	40 (30, 40)	-	25 (20, 30)	30 (25, 30)
Mode, units	90	40	-	20	30
PM+TM (5 patients, 11 treatment sessions)					
Median (Q1, Q3), units	120 (100, 200)	75 (50, 100)	-	50 (40, 100)	-
Mode, units	100	100	-	100	-
PM+SS (5 patients, 10 treatment sessions)					
Median (Q1, Q3), units	75 (60, 75)	50 (40, 50)	-	-	25 (25, 25)
Mode, units	75	50	-	-	25
LD alone (3 patients, 5 treatment sessions)					
Median (Q1, Q3), units	75 (50, 100)	-	50 (50, 60)	-	-
Mode, units	50	-	50	-	-
LD+TM (2 patients, 3 treatment sessions)					
Median (Q1, Q3), units	75 (75, 150)	-	50 (50, 50)	25 (25, 50)	-
Mode, units	75	-	50	25	-

LD: latissimus dorsi; PM: pectoralis major; Q1: first quartile; Q3: third quartile; SS: subscapularis; TM: teres major.

The table shows the combinations of muscles treated in more than one patient. The total dose may include doses injected in shoulder muscles not indicated in the table.

Table SVI. *Frequently treated combinations of elbow muscles (100 patients, 366 treatment sessions)*

	Total dose	BB dose	B dose	BR dose
BB+B (47 patients, 115 treatment sessions)				
Median (Q1, Q3), units	100 (80, 125)	50 (50, 60)	50 (30, 50)	-
Mode, units	100	50	50	-
BB alone (44 patients, 124 treatment sessions)				
Median (Q1, Q3), units	75 (50, 100)	50 (50, 95)	-	-
Mode, units	50	50	-	-
BB+B+BR (29 patients, 74 treatment sessions)				
Median (Q1, Q3), units	125 (100, 150)	50 (45, 75)	45 (25, 50)	30 (25, 50)
Mode, units	150	50	50	25
BB+BR (8 patients, 11 treatment sessions)				
Median (Q1, Q3), units	100 (90, 150)	50 (50, 100)	-	50 (40, 50)
Mode, units	150	50	-	50
B alone (3 patients, 7 treatment sessions)				
Median (Q1, Q3), units	70 (70, 100)	-	70 (50, 70)	-
Mode, units	70	-	70	-

B: brachialis; BB: biceps brachii; BR: brachioradialis; Q1: first quartile; Q3: third quartile.

The table shows the combinations of muscles treated in more than one patient. The total dose may include the dose injected in another elbow muscle (triceps brachii).

Table SVII. *Frequently treated combinations of forearm pronators (54 patients, 159 treatment sessions)*

	Total dose	PT dose	PQ dose
PT alone (53 patients, 151 treatment sessions)			
Median (Q1, Q3), units	40 (25, 50)	40 (25, 50)	-
Mode, units	50	50	-
PT+PQ (3 patients, 8 treatment sessions)			
Median (Q1, Q3), units	100 (75, 125)	62.5 (50, 87.5)	25 (25, 50)
Mode, units	75	50	25

PQ: pronator quadratus; PT: pronator teres; Q1: first quartile; Q3: third quartile.

Table SVIII. *Frequently treated combinations of wrist muscles (93 patients, 317 treatment sessions)*

	Total dose	FCR dose	FCU dose
FCR+FCU (57 patients, 159 treatment sessions)			
Median (Q1, Q3), units	100 (75, 100)	50 (45, 50)	50 (30, 50)
Mode, units	100	50	50
FCR alone (55 patients, 138 treatment sessions)			
Median (Q1, Q3), units	50 (50, 50)	50 (50, 50)	-
Mode, units	50	50	-
FCU alone (5 patients, 12 treatment sessions)			
Median (Q1, Q3), units	25 (25, 27.5)	-	25 (25, 27.5)
Mode, units	25	-	25

FCR: flexor carpi radialis; FCU: flexor carpi ulnaris; Q1: first quartile; Q3: third quartile.

The total dose may include the dose injected in another wrist muscle (palmaris longus).

Table SIX. *Frequently treated combinations of finger muscles (101 patients, 358 treatment sessions)*

	Total dose	FDS dose	FDP dose	LUM dose
FDS alone (52 patients, 149 treatment sessions)				
Median (Q1, Q3), units	50 (50, 50)	50 (50, 50)	-	-
Mode, units	50	50	-	-
FDS+FDP (48 patients, 154 treatment sessions)				
Median (Q1, Q3), units	100 (80, 125)	50 (50, 75)	50 (40, 50)	-
Mode, units	100	50	50	-
FDS+LUM (13 patients, 31 treatment sessions)				
Median (Q1, Q3), units	75 (50, 100)	40 (25, 50)	-	50 (10, 50)
Mode, units	75	40	-	50
FDP alone (5 patients, 11 treatment sessions)				
Median (Q1, Q3), units	50 (50, 50)	-	50 (50, 50)	-
Mode, units	50	-	50	-
LUM alone (5 patients, 9 treatment sessions)				
Median (Q1, Q3), units	40 (25, 50)	-	-	40 (25, 50)
Mode, units	25	-	-	25
FDS+FDP+LUM (3 patients, 4 treatment sessions)				
Median (Q1, Q3), units	95 (82.5, 100)	50 (37.5, 50)	25 (22.5, 25)	25 (22.5, 25)
Mode, units	100	50	25	25

FDP: flexor digitorum profundus; FDS: flexor digitorum superficialis; LUM: lumbricals; Q1: first quartile; Q3: third quartile.

The total dose may include doses injected in finger muscles not indicated in the table.

Table SX. *Frequently treated combinations of thumb muscles (73 patients, 223 treatment sessions)*

	Total dose	FPL dose	AP dose	FPB dose	OP dose
FPL alone (54 patients, 141 treatment sessions)					
Median (Q1, Q3), units	25 (25, 50)	25 (25, 50)	-	-	-
Mode, units	50	50	-	-	-
AP alone (18 patients, 36 treatment sessions)					
Median (Q1, Q3), units	45 (22.5, 50)	-	45 (22.5, 50)	-	-
Mode, units	50	-	50	-	-
FPL+AP (14 patients, 29 treatment sessions)					
Median (Q1, Q3), units	40 (30, 45)	20 (15, 30)	15 (15, 20)	-	-
Mode, units	30	20	15	-	-
AP+OP (4 patients, 7 treatment sessions)					
Median (Q1, Q3), units	40 (30, 40)	-	10 (10, 20)	-	20 (20, 30)
Mode, units	40	-	10	-	20
FPB alone (3 patients, 3 treatment sessions)					
Median (Q1, Q3), units	25 (10, 50)	-	-	25 (10, 50)	-
Mode, units	-	-	-	-	-
FPL+OP (2 patients, 4 treatment sessions)					
Median (Q1, Q3), units	50 (50, 50)	35 (30, 35)	-	-	15 (15, 20)
Mode, units	50	35	-	-	15

AP: adductor pollicis; FPB: flexor pollicis brevis; FPL: flexor pollicis longus; OP: opponens pollicis; Q1: first quartile; Q3: third quartile.
The table shows the combinations of muscles treated in more than one patient.

Table SXI. *Frequently treated combinations of hip muscles (16 Patients, 47 Treatment Sessions)*

	Total dose	AL dose	AM dose
AL alone (7 patients, 20 treatment sessions)			
Median (Q1, Q3), units	40 (27.5, 75)	35 (25, 55)	-
Mode, units	30	25	-
AM alone (6 patients, 11 treatment sessions)			
Median (Q1, Q3), units	50 (50, 100)	-	50 (50, 100)
Mode, units	50	-	50

AL: adductor longus; AM: adductor magnus; Q1: first quartile; Q3: third quartile.

The table shows the combinations of muscles treated in more than one patient. The total dose may include doses injected in hip muscles not indicated in the table.

Table SXII. *Frequently treated combinations of knee muscles (35 patients, 124 treatment sessions)*

	Total dose	BF dose	ST dose	SM dose	RF dose
ST+SM (15 patients, 28 treatment sessions)					
Median (Q1, Q3), units	100 (60, 100)	-	50 (30, 50)	50 (30, 50)	-
Mode, units	100	-	50	50	-
BF alone (10 patients, 26 treatment sessions)					
Median (Q1, Q3), units	75 (50, 100)	75 (50, 100)	-	-	-
Mode, units	50	50	-	-	-
BF+ST (9 patients, 16 treatment sessions)					
Median (Q1, Q3), units	100 (100, 100)	50 (50, 50)	50 (50, 50)	-	-
Mode, units	100	50	50	-	-
BF+SM (8 patients, 16 treatment sessions)					
Median (Q1, Q3), units	75 (50, 100)	38 (25, 45)	-	33.5 (25, 45)	-
Mode, units	50	25	-	25	-
BF+ST+SM (7 patients, 10 treatment sessions)					
Median (Q1, Q3), units	150 (150, 150)	50 (50, 50)	50 (50, 50)	50 (50, 50)	-
Mode, units	150	50	50	50	-
ST alone (6 patients, 8 treatment sessions)					
Median (Q1, Q3), units	50 (50, 62.5)	-	50 (50, 62.5)	-	-
Mode, units	50	-	50	-	-
SM alone (5 patients, 8 treatment sessions)					
Median (Q1, Q3), units	50 (30, 50)	-	-	50 (30, 50)	-
Mode, units	50	-	-	50	-
RF alone (2 patients, 3 treatment sessions)					
Median (Q1, Q3), units	50 (25, 50)	-	-	-	50 (25, 50)
Mode, units	50	-	-	-	50
BF+SM+RF (2 patients, 2 treatment sessions)					
Median (Q1, Q3), units	102.5 (55, 150)	35 (20, 50)	-	37.5 (25, 50)	30 (10, 50)
Mode, units	-	-	-	-	-

BF: biceps femoris; Q1: first quartile; Q3: third quartile; RF: rectus femoris; SM: semimembranosus; ST: semitendinosus.

The table shows the combinations of muscles treated in more than one patient.

Table SXIII. *Frequently treated combinations of ankle muscles (105 patients, 399 treatment sessions)*

	Total dose	GC dose	SOL dose	TP dose	TA dose
GC+SOL+TP (61 patients, 178 treatment sessions)					
Median (Q1, Q3), units	250 (200, 270)	100 (70, 120)	75 (50, 100)	50 (50, 75)	-
Mode, units	250	100	50	50	-
GC+SOL (32 patients, 94 treatment sessions)					
Median (Q1, Q3), units	200 (150, 200)	100 (100, 125)	75 (50, 100)	-	-
Mode, units	200	100	100	-	-
GC alone (14 patients, 27 treatment sessions)					
Median (Q1, Q3), units	100 (50, 100)	100 (50, 100)	-	-	-
Mode, units	100	100	-	-	-
GC+TP (13 patients, 26 treatment sessions)					
Median (Q1, Q3), units	150 (100, 200)	100 (75, 100)	-	62.5 (50, 100)	-
Mode, units	100	100	-	50	-
SOL alone (9 patients, 15 treatment sessions)					
Median (Q1, Q3), units	100 (50, 100)	-	100 (50, 100)	-	-
Mode, units	100	-	100	-	-
SOL+TP (9 patients, 13 treatment sessions)					
Median (Q1, Q3), units	125 (100, 160)	-	75 (50, 100)	50 (50, 80)	-
Mode, units	100	-	50	50	-
TP alone (7 patients, 12 treatment sessions)					
Median (Q1, Q3), units	80 (40, 130)	-	-	80 (40, 130)	-
Mode, units	130	-	-	130	-
GC+SOL+TP+TA (6 patients, 13 treatment sessions)					
Median (Q1, Q3), units	170 (155, 200)	40 (30, 75)	50 (50, 60)	40 (25, 50)	30 (25, 50)
Mode, units	170	25	50	50	25
GC+TP+TA (5 patients, 14 treatment sessions)					
Median (Q1, Q3), units	200 (190, 225)	100 (100, 100)	-	55 (50, 100)	37.5 (25, 50)
Mode, units	200	100	-	50	25
SOL+TP+TA (2 patients, 3 treatment sessions)					
Median (Q1, Q3), units	200 (90, 200)	-	70 (30, 80)	100 (30, 100)	30 (20, 30)
Mode, units	200	-	-	100	30
TP+TA (2 patients, 2 treatment sessions)					
Median (Q1, Q3), units	95 (90, 100)	-	-	45 (40, 50)	50 (50, 50)
Mode, units	-	-	-	-	50

GC: gastrocnemius; Q1: first quartile; Q3: third quartile; SOL: soleus; TA: tibialis anterior; TP: tibialis posterior.

The table shows the combinations of muscles treated in more than one patient.

Table SXIV. *Frequently treated combinations of toe muscles (71 patients, 223 treatment sessions)*

	Total dose	FDL dose	FHL dose	EHL dose
FDL alone (43 patients, 110 treatment sessions)				
Median (Q1, Q3), units	50 (30, 50)	50 (30, 50)	-	-
Mode, units	50	50	-	-
FDL+FHL (35 patients, 75 treatment sessions)				
Median (Q1, Q3), units	75 (50, 100)	50 (30, 50)	25 (20, 50)	-
Mode, units	50	50	50	-
FHL alone (11 patients, 16 treatment sessions)				
Median (Q1, Q3), units	50 (22.5, 50)	-	50 (22.5, 50)	-
Mode, units	50	-	50	-
EHL alone (5 patients, 6 treatment sessions)				
Median (Q1, Q3), units	27.5 (25, 30)	-	-	27.5 (25, 30)
Mode, units	25	-	-	25
FDL+EHL (3 patients, 4 treatment sessions)				
Median (Q1, Q3), units	125 (112.5, 157.5)	75 (62.5, 82.5)	-	50 (50, 60)
Mode, units	125	75	-	50

EHL: extensor hallucis longus; FDL: flexor digitorum longus; FHL: flexor hallucis longus; Q1: first quartile; Q3: third quartile.

The total dose may include doses injected in toe muscles not indicated in the table.

Table SXV. *Adverse events across dose ranges*

	Total (N=107)	≤400 units (N=8)	401-450 units (N=20)	451-500 units (N=42)	501-550 units (N=16)	551-600 units (N=69)
Any event	49 (45.8%)	2 (25.0%)	9 (45.0%)	24 (57.1%)	2 (12.5%)	19 (27.5%)
COVID-19	10 (9.3%)		1 (5.0%)	4 (9.5%)		5 (7.2%)
Nasopharyngitis	7 (6.5%)	1 (12.5%)	2 (10.0%)	3 (7.1%)		1 (1.4%)
Cellulitis	4 (3.7%)			2 (4.8%)		2 (2.9%)
Periarthritis	3 (2.8%)			2 (4.8%)		1 (1.4%)
Cerebral haemorrhage	2 (1.9%)			2 (4.8%)		
Cerebral infarction	2 (1.9%)		1 (5.0%)	1 (2.4%)		
Vertigo positional	2 (1.9%)		1 (5.0%)	1 (2.4%)		
Eczema	2 (1.9%)					2 (2.9%)
Back pain	2 (1.9%)		1 (5.0%)			1 (1.4%)
Asthenia	2 (1.9%)		1 (5.0%)			1 (1.4%)
Pyrexia	2 (1.9%)				1 (6.3%)	1 (1.4%)
Femoral neck fracture	2 (1.9%)			1 (2.4%)		1 (1.4%)
Contusion	2 (1.9%)		1 (5.0%)	1 (2.4%)		

Those observed in more than one case are shown here.

Some patients were included in more than one dose category as they repeated injections, resulting in the total number of patients exceeding 107, the actual count of unique patients.

Table SXVI. *Adverse events of special interest observed across treatment cycles*

	Total (N=107)	Treatment Cycle 1 (N=107)	Treatment Cycle 2 (N=106)	Treatment Cycle 3 (N=95)	Treatment Cycle 4 (N=80)
Identified as PDSOT	4 (3.7%)	1 (0.9%)	0 (0.0%)	3 (3.2%)	1 (1.3%)
Pneumonia aspiration	1 (0.9%)	1 (0.9%)		1 (1.1%)	
Constipation	1 (0.9%)				1 (1.3%)
Muscular weakness	1 (0.9%)			1 (1.1%)	
Urinary retention	1 (0.9%)			1 (1.1%)	
Related to falls and other accidents	8 (7.5%)	4 (3.7%)	1 (0.9%)	1 (1.1%)	2 (2.5%)
Femoral neck fracture	2 (1.9%)		1 (0.9%)		1 (1.3%)
Contusion	2 (1.9%)	2 (1.9%)			
Fracture	1 (0.9%)	1 (0.9%)			
Humerus fracture	1 (0.9%)		1 (0.9%)		
Shunt occlusion	1 (0.9%)	1 (0.9%)			
Spinal column injury	1 (0.9%)				1 (1.3%)
Post-traumatic neck syndrome	1 (0.9%)			1 (1.1%)	
Related to lung function	2 (1.9%)	1 (0.9%)	0 (0.0%)	1 (1.1%)	1 (1.3%)
Pneumonia aspiration	1 (0.9%)	1 (0.9%)		1 (1.1%)	
Cough	1 (0.9%)				1 (1.3%)

PDSOT: possible distant spread of toxin.

Table SXVII. *Changes in percutaneous oxygen saturation (SpO_2) across treatment cycles*

	Treatment Cycle 1 (n=60)	Treatment Cycle 2 (n=70)	Treatment Cycle 3 (n=57)	Treatment Cycle 4 (n=47)
Before treatment, %				
Mean (SD)	97.5 (1.16)	97.5 (1.10)	97.8 (1.06)	97.2 (1.13)
Median (Min, Max)	98 (94, 99)	98 (95, 99)	98 (94, 99)	97 (94, 99)
After treatment, %				
Mean (SD)	97.4 (1.19)	97.4 (1.18)	97.6 (1.07)	97.3 (1.17)
Median (Min, Max)	97 (93, 100)	97 (93, 99)	98 (94, 99)	98 (93, 99)
Change, %				
Mean (SD)	-0.1 (1.19)	-0.1 (1.25)	-0.2 (1.17)	0.1 (1.15)
Median (Min, Max)	0 (-2, 3)	0 (-4, 3)	0 (-4, 3)	0 (-3, 3)

Max: maximum; Min: minimum; SD: standard deviation.

Patients with both pre- and post-treatment values were included in the analysis for each treatment cycle.
